# Supplementary material for: A qualitative insight into researchers’ perceptions of gender inequality in medical and dental research institutions in Nigeria
Source: PLoS One. 2023 Apr 5;18(4):e0283756. doi: 10.1371/journal.pone.0283756 (PMC10075448; doi:10.1371/journal.pone.0283756)
Supplement: S1 Table — (DOCX) [file pone.0283756.s001.docx]

Supplemental File 1: Distribution of proposed 54 interviews by gender, Rank and Profession

| **Name of school** | **Gender** | **Ranks** | **Profession** |
| --- | --- | --- | --- |
| Obafemi Awolowo University | Female  Female  Male | Reader  Senior lecturer  Lecturer 1 | Medicine  Medicine  Dentist |
| University of Ibadan | Female  Female  Male | Senior lecture  Senior lecturer  Professor | Medicine  Medicine  Medicine |
| University of Lagos | Female  Female  Male | Lecturer 1  Lecturer 1  Reader | Medicine  Medicine  Medicine |
| LASUTH | Female  Female  Male | Professor  Reader  Professor | Medicine  Dentist  Medicine |
| University of Benin | Female  Female  Male | Reader  Lecturer 2  Reader | Medicine  Dentist  Dentist |
| University of Nsukka, Enugu | Female  Female  Male | Lecturer 2  Senior lecturer  Senior lecturer | Medicine  Medicine  Dentist |
| University of Maiduguri | Female  Female  Male | Senior lecturer  Professor  Reader | Medicine  Medicine  Medicine |
| University of Sokoto | Female  Female  Male | Reader  Reader  Lecturer 1 | Dentist Dentist  Dentist |
| University of Jos | Female  Female  Male | Senior lecturer  Senior lecturer  Senior lecturer | Dentist  Medicine  Dentist |
| Bayero University Kano | Female  Female  Male | Lecturer 1  Lecturer 2  Lecturer 1 | Medicine  Dentist  Medicine |
| Afe Babalola | Female  Female  Male | Senior lecturer  Lecturer 1  Senior lecturer | Dentist  Dentist  Medicine |
| University of Port-harcourt | Female  Female  Male | Professor  Professor  Lecturer 2 | Dentist  Medicine  Medicine |
| University of Calabar | Female  Female  Male | Lecturer 1  Reader  Lecturer 1 | Medicine  Medicine  Medicine |
| Unimed, Ondo | Female  Female  Male | Lecturer 1  Lecturer 1  Senior lecturer | Dentist  Medicine  Medicine |
