# Supplementary material for: A qualitative insight into researchers’ perceptions of gender inequality in medical and dental research institutions in Nigeria
Source: PLoS One. 2023 Apr 5;18(4):e0283756. doi: 10.1371/journal.pone.0283756 (PMC10075448; doi:10.1371/journal.pone.0283756)
Supplement: Attachment — Submitted filename: Reviewers response_Plos One 3.0.docx [file pone.0283756.s002.docx]

We will like to thank the reviewers and the editor for the thorough review of this manuscript. It has helped improved the quality of the research.

1. Please ensure that your manuscript meets PLOS ONE's style requirements, including those for file naming. The PLOS ONE style templates can be found at
   https://journals.plos.org/plosone/s/file?id=wjVg/PLOSOne_formatting_sample_main_body.pdf and 
   https://journals.plos.org/plosone/s/file?id=ba62/PLOSOne_formatting_sample_title_authors_affiliations.pdf

Response: this has been checked and revised accordingly

2. We note that the grant information you provided in the ‘Funding Information’ and ‘Financial Disclosure’ sections do not match. When you resubmit, please ensure that you provide the correct grant numbers for the awards you received for your study in the ‘Funding Information’ section."

Response: This has been corrected. No grant was received for the study

3. Thank you for stating the following financial disclosure: 
"MOF received funding to conduct the data collection and analysis"

At this time, please address the following queries:

a) Please clarify the sources of funding (financial or material support) for your study. List the grants or organizations that supported your study, including funding received from your institution.

Response: This has been corrected. The authors received no specific funding for this work

b) State what role the funders took in the study. If the funders had no role in your study, please state: “The funders had no role in study design, data collection and analysis, decision to publish, or preparation of the manuscript.”

Response: This has been corrected. The authors received no specific funding for this work

c) If any authors received a salary from any of your funders, please state which authors and which funders.

Response: This has been corrected. The authors received no specific funding for this work

d) If you did not receive any funding for this study, please state: “The authors received no specific funding for this work.”

Response: This has been corrected. The authors received no specific funding for this work

Please include your amended statements within your cover letter; we will change the online submission form on your behalf.

Response: this has been done

4. Thank you for stating the following in the Acknowledgments Section of your manuscript: 
"The authors acknowledge afworo that provided funding support for the data collection and analysis of this piece of work."

Response: This has been corrected. The authors received no specific funding for this work

We note that you have provided funding information that is not currently declared in your Funding Statement. However, funding information should not appear in the Acknowledgments section or other areas of your manuscript. We will only publish funding information present in the Funding Statement section of the online submission form.

Please remove any funding-related text from the manuscript and let us know how you would like to update your Funding Statement. Currently, your Funding Statement reads as follows: 
"MOF received funding to conduct the data collection and analysis"

Response: This has been corrected. The authors received no specific funding for this work

Please include your amended statements within your cover letter; we will change the online submission form on your behalf.

Response: This has been corrected. The authors received no specific funding for this work

5. In your Data Availability statement, you have not specified where the minimal data set underlying the results described in your manuscript can be found. PLOS defines a study's minimal data set as the underlying data used to reach the conclusions drawn in the manuscript and any additional data required to replicate the reported study findings in their entirety. All PLOS journals require that the minimal data set be made fully available. For more information about our data policy, please see http://journals.plos.org/plosone/s/data-availability.

Response: The data needed for this study can all be found in the study result section

Upon re-submitting your revised manuscript, please upload your study’s minimal underlying data set as either Supporting Information files or to a stable, public repository and include the relevant URLs, DOIs, or accession numbers within your revised cover letter. For a list of acceptable repositories, please see http://journals.plos.org/plosone/s/data-availability#loc-recommended-repositories. Any potentially identifying patient information must be fully anonymized.

Response: The data needed for this study can all be found in the study result section

Important: If there are ethical or legal restrictions to sharing your data publicly, please explain these restrictions in detail. Please see our guidelines for more information on what we consider unacceptable restrictions to publicly sharing data: http://journals.plos.org/plosone/s/data-availability#loc-unacceptable-data-access-restrictions. Note that it is not acceptable for the authors to be the sole named individuals responsible for ensuring data access.

Response: The data needed for this study can all be found in the study result section

We will update your Data Availability statement to reflect the information you provide in your cover letter.

Response: The data needed for this study can all be found in the study result section

Reviewer #1: • Add more papers from 2017 to 2022 in the introduction section. It should be motivated for the reader and also add unique contribution.

Response: A literature search was carried out prior to this resubmission and this elicited 4 new relevant studies that have been included in the introduction section

• Address theoretical background of the study and also add theoretical implication (Support with theories).

Response: Done. The Feminist institutionalism analysis approach was used for this study. See lines 102-110

• Author can mention objective of the study and research gap separately which enhance quality of paper.

Response: The final paragraphs have now been dedicated to a focused discussion relating back to the study objectives and research questions

• Add theoretical and managerial implications

Response: Thank you for raising this useful point. Lines 425 to 442 expand on this issue

• Explain conclusion, limitation and future scope separately.

Response: We have included two paragraphs that discuss the strengths, limitations, and future scope of the study. See Lines 425 to 442. A paragraph has also been added to the conclusion section

• The language of the paper needs a careful editing.

Response: The entire paper has been edited for style and language

• References must follow the style as per journal

Response: The manuscript has been revised to adhere to the style of the journal

Reviewer #2: Paper looks good and I strongly believe that this paper is contributiing to the body of Knowledge. Well done!! It is interesting well organized paper. It was pleasure to review it. Thank you for the opportunity.

Response: thank you for this positive feedback.
